# Alleviating Effects of Ethanol Extract from *Acremonium terricola* Culture on Patulin Toxicity

**DOI:** 10.3390/antiox14050509

**Published:** 2025-04-24

**Authors:** Haiyan Lin, Savindi Kaushalya Edirisinghe, Ijeoma Esther Okolo, Zhen Chen, Juan Sun, Wei Hong, Ruiyu Zhu

**Affiliations:** 1 School of Biological and Chemical Engineering, Zhejiang University of Science and Technology, Hangzhou 310023, China; linhy22@zust.edu.cn (H.L.); savindike26@zust.edu.cn (S.K.E.); 912403817006@zust.edu.cn (I.E.O.); 212103817018@zust.edu.cn (Z.C.); sunjuan18@zust.edu.cn (J.S.); 2 College of Food Science, Fujian Agriculture and Forestry University, Fuzhou 350002, China; 3 Key Laboratory of Endemic and Ethnic Diseases, Ministry of Education and Key Laboratory of Medical Molecular Biology of Guizhou Province, Guizhou Medical University, Guiyang 550004, China; hongwei@gmc.edu.cn

**Keywords:** patulin, toxicity alleviation, *Acremonium terricola*, ethanol extract, *Cordyceps gunnii*, molecular docking, UPLC-ESI-MS/MS

## Abstract

Exposure to patulin (PAT) poses a significant health risk to animals, emphasizing the need for natural, safe substances to mitigate toxicity. *Acremonium terricola* culture (ATC), a fungal fermentation-derived feed additive, is known for its antioxidant properties, yet its potential to alleviate mycotoxin-induced toxicity remains largely uninvestigated. In this study, the ethanol extracts from the ATC (EEAT) were prepared with a total phenolic content of 67.9 mg GAE/g and a total flavonoid content of 32.7 mg RE/g. Ultra-high performance liquid chromatography coupled with tandem mass spectrometry (UPLC-ESI-MS/MS) analysis was employed to investigate the bioactive components in EEAT. In PAT-exposed *Caenorhabditis elegans* models, EEAT treatment significantly enhanced locomotory capacity and elevated antioxidant enzyme activities by 63.1% (SOD) and 90.1% (GSH-ST), respectively. Molecular docking analysis revealed that key active compounds in EEAT, such as coumarin, succinic acid, and trigonelline, exhibited effective binding affinities to potential targets SIR-2.1 and DAF-2. Notably, coumarin and trigonelline were most effective in alleviating PAT toxicity, as evidenced by rescued locomotor rates and oxidative impairment in *C. elegans*. Our findings not only elucidate the molecular basis of EEAT-mediated PAT mitigation but also establish *A. terricola* culture as a sustainable antioxidant.

## 1. Introduction

Mycotoxins are common fungal secondary metabolites in cereals, fruits, and vegetables [1]. Patulin (PAT), polyketide lactone (4-hydroxy-4H-furo [3,2-c] pyran-2(6H)-one), is primarily secreted by *Penicillium*, *Aspergillus*, and *Byssochlamys* species [2,3]. Recent surveillance studies have documented varying PAT contamination levels across diverse agricultural products (e.g., fruits and fruit juice) and geographical regions (e.g., Pakistan, Qatar, China) [4,5,6]. Chronic exposure to PAT has been linked to neurotoxicity, renal toxicity, and other health risks [7], largely attributed to its capacity to deplete glutathione (GSH) reserves, inhibit glutathione S-transferase (GST) activity, and induce excessive intracellular reactive oxygen species (ROS) accumulation [8,9]. Consequently, identifying effective strategies to counteract PAT-induced oxidative damage remains a critical research priority.

Current approaches to mitigate mycotoxin toxicity emphasize dietary interventions utilizing probiotics and phytochemicals. For example, the intake of *Lactobacillus plantarum* and *Saccharomyces cerevisiae* has been reported to reduce oxidative stress induced by PAT in *C. elegans* [10,11]. However, probiotic-based approaches require strict viability control during storage and gastrointestinal transit, constraining their practical application. Similarly, plant-derived compounds such as curcumin, lycopene, and resveratrol exhibit protective effects against aflatoxin toxicity through antioxidative and detoxification pathways [12,13,14]. A recent study demonstrated apple polyphenols’ ability to alleviate PAT-induced intestinal damage via gut microbiota modulation [15]. Nevertheless, existing interventions predominantly target aflatoxins, as well as lacking PAT-specific toxicity mechanisms. The potential of natural bioactive compounds from non-plant sources, particularly fungi, in alleviating PAT toxicity remains underexplored.

Fungi have recently garnered attention as reservoirs of bioactive metabolites with detoxification potential. *Acremonium terricola*, a natural asexual fungal strain derived from *Cordyceps gunnii*, is artificially extracted to produce effective products through solid fermentation [16,17]. *A. terricola* culture (ATC) has emerged as a promising alternative to synthetic antibiotics as a feed additive, largely due to its rich composition of bioactive compounds, such as nucleosides (adenosine and cordycepin), vitamins (vitamin B), sugar alcohols (cordycepic acid), and sterols (ergosterol) [17]. Contemporary research demonstrates that these secondary metabolites exhibit potent antioxidant properties and immunoregulatory capacities in animal systems [17,18,19,20], characteristics that are particularly relevant for mycotoxin mitigation. However, the precise composition of ATC’s bioactive arsenal and its mechanistic action against PAT-induced toxicity remain poorly characterized. *Caenorhabditis elegans* has emerged as a powerful toxicological model due to its conserved detoxification pathways and physiological relevance to mammalian systems [21]. Its high-throughput capacity enables efficient screening of bioactive compounds through quantitative assessment of motility parameters, oxidative stress markers, and enzymatic activity under controlled conditions [22]. Leveraging these advantages, we employed *C. elegans* to investigate the detoxification potential of EEAT against PAT exposure, aiming to identify lead compounds for subsequent vertebrate studies.

In this study, UPLC-ESI-MS/MS provided comprehensive phytochemical profiling of EEAT. Quantitative analysis of nematode locomotion patterns and oxidative damage biomarkers served to evaluate PAT toxicity mitigation. Through both simulation assays and empirical validation, the detoxification efficacy of three key EEAT constituents was demonstrated. This research provides a sustainable and cost-effective strategy for mitigating mycotoxin toxicity by utilizing fungal fermentation-derived bioactive compounds.

## 2. Materials and Methods

### 2.1. Materials and Strains

The ATC fermentation powder was purchased from Zhejiang Esigma Biological Co., Ltd. (Haining, China). *Caenorhabditis elegans* wild-type N2 and *Escherichia coli* strain (OP50) were acquired from the *Caenorhabditis* Genetics Center (Minneapolis, MN, USA). The *Penicillium expansum* strain was obtained from the China Center for Microbiological Culture Collection (Beijing, China).

### 2.2. Equipment

The principal instrumentation employed in this investigation comprised a high-speed freezing centrifuge (Kecheng H2-16KR, Changsha, China), a vortexer (IKA Vortex 2, Staufen, Germany), a rotary evaporator (Bluepard RV-211A, Shanghai, China), a freeze-drying apparatus (SCIENTZ-10N/C, Ningbo, China), a multi-mode microplate reader (Allsheng Feyond-A300, Hangzhou, China), a shaking incubator (ChiChu ZQLY-180, Shanghai, China), biochemical incubators (Bluepard LRH-70, Shanghai, China), preparative liquid chromatography (Shimadzu LC-8A, Kyoto, Japan), a stereo microscope (Olympus SZ61, Tokyo, Japan), a fluorescence microscope (OLYMPUS IX73, Hamburg, Germany), a probe sonicator (Sonics VCX750, Newtown, CT, USA), and a UPLC-MS/MS platform (Thermo Fisher Scientific, Waltham, MA, USA).

### 2.3. Extraction of EEAT

The extraction process of EEAT was conducted following the methods reported by Wang et al. [23] with minor modifications. Briefly, the fermentation powder of *A. terricola* was sieved through a 0.25 mm mesh and extracted with anhydrous ethanol at a solid-to-liquid ratio of 1:10. The extraction was performed at 60 °C using 40 kHz ultrasonic treatment for 1 h. The supernatant was collected by centrifugation (3824× *g*, 15 min, 25 °C), and the extraction process was repeated twice. The combined supernatants were concentrated using rotary evaporation at 70 °C (50 rpm) and subsequently freeze-dried in a freeze-drying apparatus (–55 °C, 48 h) to obtain the solid powder.

The total phenolic content (TPC) of EEAT was determined using gallic acid as the standard, following a modified protocol based on Derakhshan et al. [24]. Briefly, 1 mL of EEAT (0.1 g in anhydrous ethanol) was mixed with 1 mL of Folin-Ciocalteu reagent and 3 mL of 20% (*w*/*v*) sodium carbonate solution. The mixture was incubated at 25 °C for 30 min, and the absorbance was measured at 765 nm. TPC was calculated using a gallic acid calibration curve and expressed as gallic acid equivalents (mg GAE)/g. The total flavonoid content (TFC) was quantified using rutin as the standard [25]. Specifically, 1 mL of EEAT (0.1 g in anhydrous ethanol) was mixed thoroughly with 0.3 mL of 5% sodium nitrite solution. After 5 min, 0.3 mL of 10% aluminum chloride (AlCl₃) solution was added, followed by 6 min of incubation. Then, 4 mL of 4% sodium hydroxide (NaOH) solution was added, and the mixture was allowed to stand for 15 min. The absorbance was measured at 510 nm, and TFC was expressed as rutin equivalents (mg RE/g).

### 2.4. Cultivation, Chunking, and Synchronization of C. elegans

#### 2.4.1. Cultivation and Passaging of *C. elegans*

*E. coli* (OP50) solution was spread on prepared Nematode Growth Medium (NGM) plates and dried under sterile conditions. A small piece of NGM previously used for nematode culture was cut using a spatula sterilized with an alcohol burner. This piece was gently inverted and placed onto fresh NGM medium. The cultures were incubated at 22 °C for subculturing [26].

#### 2.4.2. Synchronization of *C. elegans*

The nematodes containing eggs at the L4 stage were washed (425× *g*, 1 min, 22 °C) with K-medium solution (3 M KCl:5 M NaCl). After standing for 5 min, the supernatant was removed, and the nematodes were washed (425× *g*, 2 min, 22 °C) thrice with the K-medium solution. Then, 1 mL of nematode lysis solution (50 µg/mL 4% NaClO and 50 µg/mL 10 M NaOH) was added to the precipitated nematodes. The mixture was shaken on a vortexer to completely lyse the nematodes. The lysate was then centrifuged at 425× *g* for 3 min. The resulting precipitate contained the eggs. The eggs were thoroughly washed (425× *g*, 1 min, 22 °C) 3 times with K-medium solution and subsequently transferred to NGM medium supplemented with *E. coli* OP50 for further culturing, thereby achieving synchronization [26].

### 2.5. Preparation and Purification of PAT

*P. expansum* was inoculated into the prepared potato dextrose broth (PDB) at 25 °C for 7 days. An equal volume of ethyl acetate solution was added to extract PAT (1:1, vol:vol). The mixture was shaken at 150 rpm for 1 h, and the upper organic phase was separated with a separation funnel. The ethyl acetate was removed using a rotary evaporator maintained at 40 °C with a rotation speed of 50 rpm.

The residual was dissolved in a 10% methanol solution, filtered through a 0.22 μm organic filter membrane (Shimadzu, Japan), and purified by preparative liquid chromatography. Elution was performed on a Supersil ODS2 reversed-phase column (Elite, 10 × 250 mm, 5 μm, Dalian, China) with a mobile phase of methanol and 0.1% formic acid water (15:85, vol:vol) at a flow rate of 1.0 mL/min at room temperature. The detection wavelength was set to 276 nm [6]. The resulting powder was dissolved in ethyl acetate and stored at −20 °C.

### 2.6. Alleviating Effect of EEAT on PAT

Wild-type *C. elegans* (strain N2) were cultured on NGM plates seeded with *E. coli* OP50 at 20 °C. Synchronized L1-stage nematodes (n = 40 per dish) were divided into the following seven experimental groups: (1) Untreated control (Con); (2) Control treated with 1% DMSO (DMSO); (3) Exposure to 50 µg/mL PAT (PAT); (4) Combined treatment with 1% DMSO and 50 µg/mL PAT (DMSO + PAT); (5–7) Combined treatment with 50 µg/mL PAT and escalating concentrations of EEAT (25, 50, or 100 µg/mL) in 1% DMSO groups (EEAT + PAT). All treatments were conducted in K-medium for 48 h at 20 °C.

Locomotory behavior analysis was performed using a stereo microscope. Head swings (lateral cephalic movements per minute) and body bends (full-body sinusoidal waves per 20 s) were quantified for 15–20 randomly selected nematodes per replicate [27]. Three replicates were set up for each group, and experiments were repeated 3 times.

### 2.7. Fluorescent Staining

After synchronization, the nematodes at the L1 stage were exposed to experimental conditions, as described in Section 2.6, with the EEAT + PAT group receiving 25 µg/mL EEAT for 48 h. Subsequently, nematodes were stained with 10 µM 2′,7′-dichlorodihydrofluorescein diacetate (DCFH-DA) for ROS detection, 10 µM dihydroethidium (DHE) for O_2_^−^ generation measurement, and 40 µM naphthalene-2,3-dicarboxaldehyde (NDA) for glutathione quantification, respectively. Following 30 min of staining in darkness, nematodes were heat-inactivated (60 °C, 3 min). All samples were observed using a fluorescence microscope under the same conditions. The visual fluorescence intensity was measured by ImageJ v1.52a [10]. Three replicates were set up for each treatment group, and experiments were repeated 3 times.

### 2.8. Determination of Antioxidant Enzyme Activity

The grouping followed the method in Section 2.7. The nematodes were prepared by ultrasonic disruption for 10 min (100 W, ultrasound 2 s, and interval 2 s) in ice-cold PBS using a probe sonicator [28]. After centrifugation (12,000× *g*, 15 min, 4 °C), supernatants were analyzed for the protein concentration, superoxide dismutase (SOD), and glutathione S-transferase (GST) content according to the instructions of the commercial kits (Nanjing Jiancheng Biological Co., Ltd., Nanjing, China). Three replicates were set up for each treatment group, and experiments were repeated 3 times.

### 2.9. EEAT Full Component Analysis

For the analysis of all EEAT profiling, UPLC-MS/MS analysis was performed using a Hypersil GOLD aQ column (2.1 × 100 mm, 1.9 µm) on a Q Exactive HF system (Thermo Fisher Scientific, Waltham, MA, USA). The detection process was completed by Shenzhen BGI Co., Ltd. (Shenzhen, China). Compound Discoverer 3.1 (Thermo Fisher Scientific, Waltham, MA, USA) software was used for LC-MS/MS data processing, mainly for peak extraction, peak alignment, and compound identification. The condition of chromatography was as follows: mobile phase A was 0.1% formic acid in water, while mobile phase B was 0.1% formic acid in 100% acetonitrile. Gradient elution: 0–2 min: 95% A solution, 5% B solution; 2–22 min: from 95% to 5% A solution, from 5% to 95% B solution; 22–27 min: 5% A solution, 95% B solution; 27.1–30 min: 95% A solution, 5% B solution. The flow rate was 0.3 mL/min, the column temperature was kept at 40 °C, and the injection volume was 5 μL. Mass spectrometry conditions were carried out on a Q Exactive mass spectrometer (Thermo Fisher Scientific, Waltham, MA, USA) for primary and secondary mass spectrometry data acquisition. Metabolites with a confidence level of 1 were compounds confirmed by reference standards under identical analytical conditions.

### 2.10. Molecular Docking of EEAT Active Ingredients with Oxidative Stress Target Proteins

To simulate the mechanism of EEAT in improving oxidative stress, “*Caenorhabditis elegans*”, active ingredients of EEAT, and “antioxidant” were used as keywords. Nematode proteins with antioxidant regulatory functions as well as the capacity to respond to the active ingredients (also appearing in the EEAT component profile) were screened out through the literature retrieval and analysis. The metabolites with level 1 credibility of EEAT were used as ligand molecules, and nematode-related proteins were used as receptor macromolecules for molecular docking simulations.

The EETA ligand molecules were initially drawn using ChemDraw v22.0.0.22, followed by 3D structure construction and energy minimization (MM2) using Chem3D v22.0.0.22. The processed ligand molecules were saved in mol format. Protein crystal structures in pdb format were acquired from the RCSB Protein Data Bank (https://www.rcsb.org/, accessed 20 December 2024), while predicted structures for targets lacking experimental validation were retrieved from the AlphaFold Protein Structure Database (https://alphafold.ebi.ac.uk/, accessed 20 December 2024). Relevant protein accession numbers were cataloged in Section 3.5. Structural preparations were performed using AutoDock Tools v4.2.6 (https://autodock.scripps.edu/, accessed on 21 December 2024) under the default working directory, from which protein receptors were prepared by hydrogen addition and water molecule removal, and ligand compounds were parameterized by hydrogenation, followed by automated detection of rotatable bonds and torsional centers. All processed files were saved in pdbqt format, and the “Grid Box” parameters were determined (detailed in Table S1). Molecular docking simulations were conducted using AutoDock Vina v1.1.2 (https://vina.scripps.edu/, accessed 21 December 2024) with default parameters. Resultant binding poses were converted to pdb format via PyMOL v2.4 (https://www.pymol.org/, accessed 21 December 2024) and subjected to interaction analysis using the PLIP web platform (https://plip-tool.biotec.tu-dresden.de/, accessed 21 December 2024).

### 2.11. Experimental Validation of Docking Results

The control group (Con) and “PAT” group were cultured as described in Section 2.6. In the treatment groups, 50 µg/mL of PAT and three candidate compounds (coumarin, trigonelline, and succinic acid) at concentrations of 25, 50, and 100 µg/mL (dissolved in 1% DMSO) were added to the K-medium for 48 h. Head swing and body bending frequency were measured as described in Section 2.6.

The groups for fluorescent staining were designated as “Con”, “PAT” (50 µg/mL), “PAT + coumarin” (50 µg/mL PAT + 100 µg/mL coumarin), “PAT + trigonelline” (50 µg/mL PAT + 100 µg/mL trigonelline), and “PAT + succinic acid” (50 µg/mL PAT + 100 µg/mL succinic acid). Fluorescent staining was performed as described in Section 2.7. Three replicates were set up for each treatment group, and experiments were repeated 3 times.

### 2.12. Data Statistics and Analysis

Statistical analyses were conducted using GraphPad Prism v9.5. One-way analysis of variance analyses (ANOVA) with Tukey’s honest significant difference (Tukey’s HSD) test was employed to evaluate group differences, where statistical significance was established at *p* < 0.05.

## 3. Results

### 3.1. Alleviating Effects of EEAT on Locomotory Capacity Impairment Induced by PAT in C. elegans

**Figure 1:**
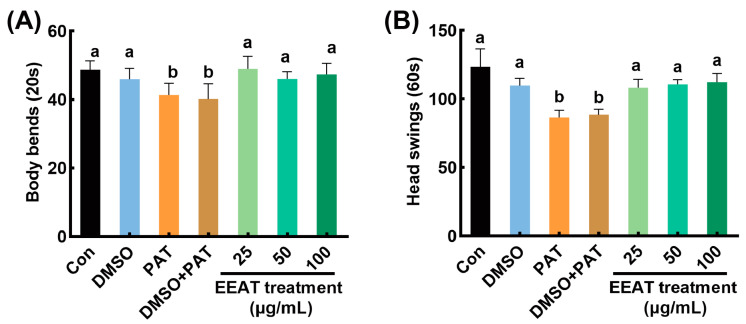
Alleviating effect of EEAT on the toxicity of PAT in *C. elegans*. The frequency of body bends (**A**) and head swings (**B**) of *C. elegans* under different treatments. “Con” referred to the control group, “DMSO” referred to *C. elegans* treated with 1% DMSO, and “PAT” referred to the model group treated with 50 µg/mL PAT. “DMSO + PAT” referred to *C. elegans* treated with 1% DMSO and 50 µg/mL PAT. “EEAT treatment” referred to groups treated with 50 µg/mL PAT and EEAT (with concentrations of 25, 50, and 100 µg/mL). Means with different letters within the same row differed significantly (*p* < 0.05).

As shown in Figure 1, the body bending and head swing frequency of the nematodes in the blank control group were 48.7 ± 0.6 and 122.3 ± 1.3, respectively. PAT exposure significantly reduced these metrics by 16% and 30%, respectively, indicating substantial neuromuscular toxicity.

Upon the application of EEAT in the PAT toxicity model, the decline in nematode locomotory capacity was significantly alleviated. Among the treatments with varying concentrations of EEAT, the body bending frequency was maximized at 25 µg/mL of EEAT, showing a 15% increase compared to the PAT group. The head swing frequency (112.2 ± 1.8) reached its highest value at 100 µg/mL of EEAT, a value not significantly different from the control group. Despite these, no significant differences were found in the alleviating effects of EEAT with different concentrations on PAT-induced toxicity. Notably, the solvent control (1% DMSO) exerted no significant effects on locomotion parameters in either untreated or PAT-exposed nematodes.

### 3.2. Alleviating Effects of EEAT on Oxidative Damage Induced by PAT in C. elegans

**Figure 2:**
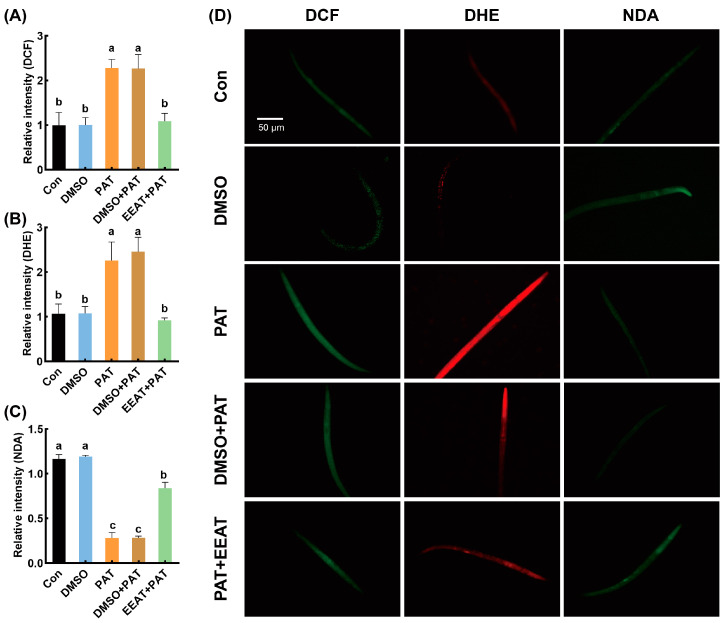
Alleviating effects of EEAT on oxidative damage induced by PAT in *C. elegans*. The relative fluorescence intensity quantification of *C. elegans* stained by DCF (**A**), DHE (**B**), and NDA (**C**) probes, and the representative images of the fluorescence staining observation (**D**). “Con” referred to the control group, “DMSO” referred to *C. elegans* treated with 1% DMSO, and “PAT” referred to the model group treated with 50 µg/mL PAT. “DMSO + PAT” referred to *C. elegans* treated with 1% DMSO and 50 µg/mL PAT. “EEAT + PAT” referred to groups treated with 50 µg/mL PAT and 25 µg/mL EEAT. Means with different letters within the same row differ significantly (*p* < 0.05).

PAT-induced oxidative stress was evidenced by 2.2-fold and 2.1-fold increases in DCF (ROS) and DHE (O_2_^−^) fluorescence intensities, respectively (Figure 2A,B,D). In the treatment of PAT + EEAT, fluorescence intensity was significantly reduced, showing no significant difference compared to the control group.

The NDA fluorescence intensity of nematodes in the control group was strong, whereas it was significantly reduced in the PAT-treated group, indicating that PAT caused a reduction in GSH levels in the nematodes. In the PAT + EEAT treatment group, the green fluorescence intensity was restored to levels comparable to the control group, with no significant difference observed (Figure 2C,D). Consistent with previous observations, 1% DMSO showed no modulatory effects on redox biomarkers in either the control or PAT-treated groups.

### 3.3. Effects of EEAT on Antioxidant Enzyme Activity Inhibited by PAT in C. elegans

**Figure 3:**
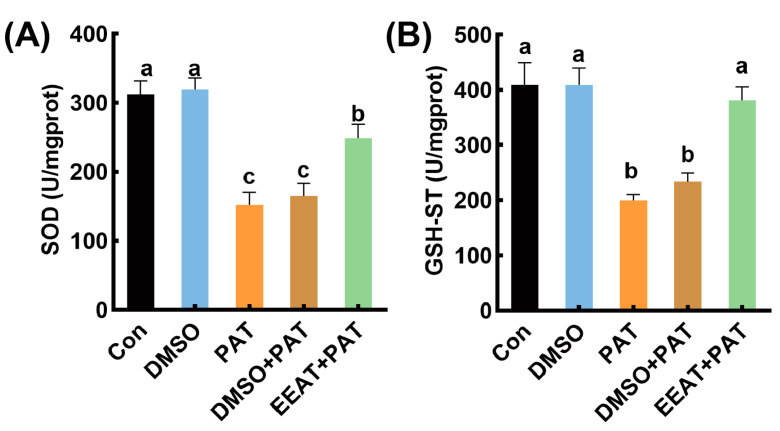
The antioxidant oxidase activities in *C. elegans* under different treatments. (**A**) SOD activity; (**B**) GSH-ST enzyme activity. The grouping information was consistent with the grouping in Figure 2. Means with different letters within the same row differ significantly (*p* < 0.05).

As shown in Figure 3, the SOD and GSH-ST enzyme activities in nematodes exposed to PAT were significantly reduced by 37.82% and 52.81%, respectively, compared to the control group. In the PAT + EEAT treatment group, the activities of SOD and GSH-ST were significantly improved by 63.1 and 90.1%, respectively, compared to the PAT group. The 1% DMSO solvent had no significant inhibitory effect on either SOD or GSH-ST enzyme activities, nor did it alleviate the inhibitory effects of PAT.

### 3.4. The Chemical Composition Analysis of EEAT

The contents of total phenols and total flavonoids in EEAT were 67.9 ± 1.2 mg GAE/g and 32.7 ± 0.7 mg RE/g, respectively. The UPLC-ESI-MS/MS analysis method was successfully applied to the analysis of chemical composition in the ethanol extract of ATC. In the negative ion mode (Figure S1A), a total of 4031 compounds were detected, of which 1496 were identified. Metabolites with a level 1 confidence were screened out as shown in Table 1, including 3 amino acids (lysine, proline, and leucine), 2 nucleosides (uracil and adenine), and 4 organic acids (succinic acid, shikimic acid, citric acid, and quinic acid). In the positive ion mode (Figure S1B), a total of 8228 compounds were detected. The same screening method as in the positive ion mode was used to screen out 16 compounds, including 4 amino acids (isoleucine, proline, phenylalanine, and L-norleucine), 2 nucleosides (guanine and adenine), 2 alkaloids (acetylcholine and trigonelline), 3 organic acids (2,2-dimethyl succinic acid, 4-guanidino butyric acid, and jasmonic acid), and 1 sugar alcohol (galactitol). In addition, D-fructose-1,6-diphosphate sodium, coumarin, cortisol, and bilirubin were also found (Table 2).

### 3.5. Docking of EEAT Active Ingredients and Target Proteins

**Figure 4:**
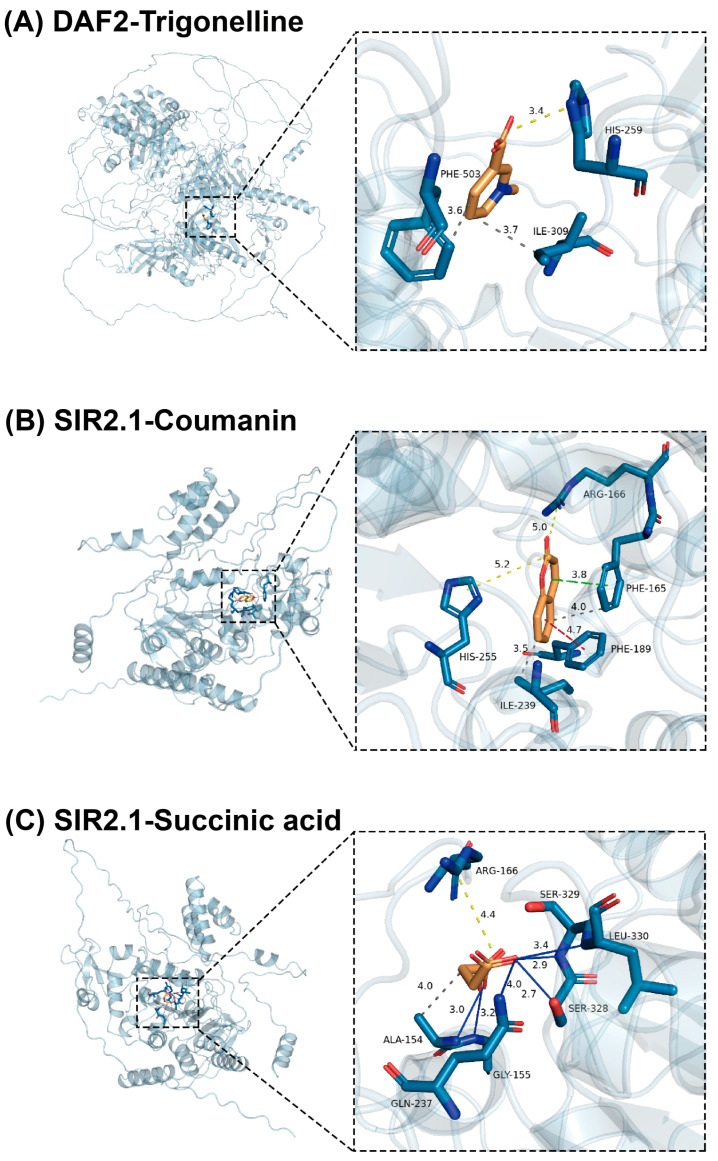
Visualization of optimal molecular docking results of three compounds. The docking results of receptor DAF-2 with ligand trigonelline (**A**), receptor SIR2.1 with ligand coumarin (**B**), and succinic acid (**C**), respectively. The dotted grey lines represent hydrophobic interactions, the dotted yellow lines represent salt bridges, the dotted green lines represent π-stacking (parallel), the dotted red lines represent π-stacking (perpendicular), and the solid blue lines represent hydrogen bonds.

Based on the literature retrieval and analysis results, coumarin, trigonelline, and succinic acid were selected as ligand small molecules. Meanwhile, 14 antioxidant-related proteins in *C. elegans* were chosen, including SKN-1, SOD-3, GST-4, HSP-1, DAF-16, HSF-1, DAF-2, AGE-1, GLP-1, AAK-2, ATFS-1, UBL-5, SIR-2.1, and EAT-2. Molecular docking results showed strong binding affinities (ranging from −3.3 to −7.0 kcal/mol) between 3 ligands and 14 receptors (Table 3). Notably, coumarin and succinic acid exhibited the strongest binding affinity for the SIR-2.1 protein, while trigonelline showed the strongest binding to the DAF-2 protein. Structural visualization of these three ligand-receptor systems elucidated their distinct interaction patterns (Figure 4).

As detailed in Figure 4A, trigonelline establishes an ionic interaction with His259 of DAF-2 at a bond distance of 3.4 Å. Concurrently, hydrophobic interactions are observed between ligands with Ile309 (3.7 Å) and Phe503 (3.6 Å). The binding energy shift is partially attributed to entropic gains from desolvation-driven water displacement during complex formation. These synergistic non-covalent forces markedly improve trigonelline-DAF-2 binding affinity through energetic stabilization and reduced binding free energy, ultimately yielding a stable complex. In Figure 4B, coumarin forms ionic bonds with residues Arg166 (5.0 Å) and His255 (5.2 Å) of protein SIR2.1. The ligand engages in π-π stacking interactions with Phe165 (3.8 Å, parallel conformation) and Phe189 (4.7 Å, T-shaped conformation) [29]. Additionally, ionic bonds are formed with Phe165 (4.0 Å) and Ile239 (3.5 Å). These interactions not only strengthen the binding stability between coumarin and SIR2.1 but also contribute to maintaining the protein’s structural integrity. As demonstrated in Figure 4C, succinic acid forms an ionic bond with residue Arg166 of SIR2.1 at 4.4 Å and hydrophobic interactions with Ala154 (4.0 Å). Hydrogen bonds are formed between ligands and residues Ala154 (3.0 Å), Gly155 (3.5 Å), Gln237 (4.0 Å), Ser328 (2.7 Å), Ser329 (2.9 Å), and Leu330 (3.4 Å). Based on hydrogen bond length criteria [30], moderate hydrogen bonds (2.5–3.2 Å) were observed between ligands and residues Ser328 (2.7 Å), Ser329 (2.9 Å), and Ala154 (3.0 Å). Weak hydrogen bonds (>3.2 Å) were observed between ligands and residues Leu330 (3.4 Å), Gly155 (3.5 Å), and Gln237 (4.0 Å). It is noteworthy that all hydrogen bond angles fall within normal ranges [31], thus validating the reliability of the docking process. Collectively, these interactions ensure tight binding between succinic acid and SIR2.1, thereby forming a stable complex.

### 3.6. Verification of Molecular Docking Results

**Figure 5:**
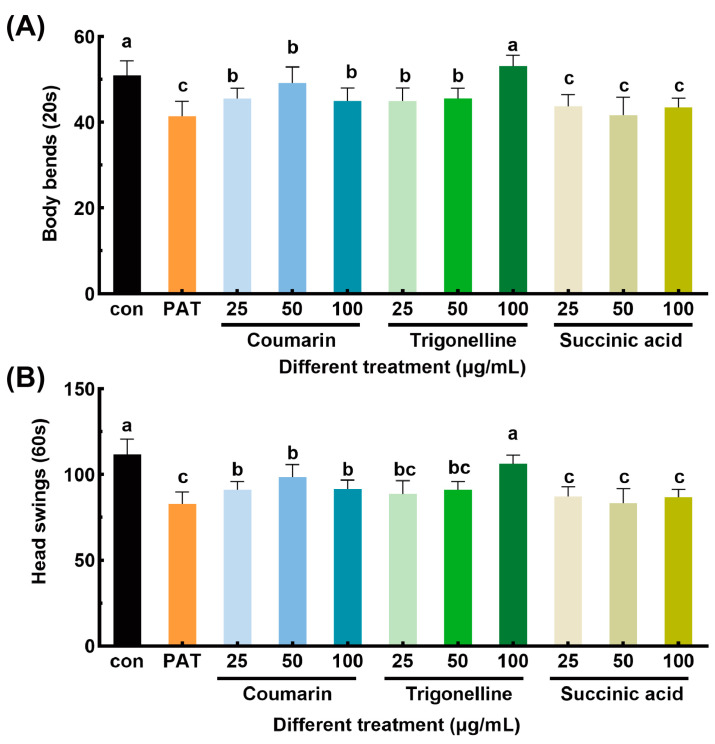
Alleviating effects of 3 active substances on the toxicity of PAT in *C. elegans*. The frequency of body bends (**A**) and head swings (**B**) of *C. elegans* under different treatments. “Con” referred to the control group, and “PAT” referred to the model group treated with 50 µg/mL PAT. “Coumarin”, “Trigonelline”, and “Succinic acid” referred to groups treated with 50 µg/mL PAT and corresponding compounds with concentrations of 25, 50, and 100 µg/mL, respectively. Means with different letters within the same row differed significantly (*p* < 0.05).

**Figure 6:**
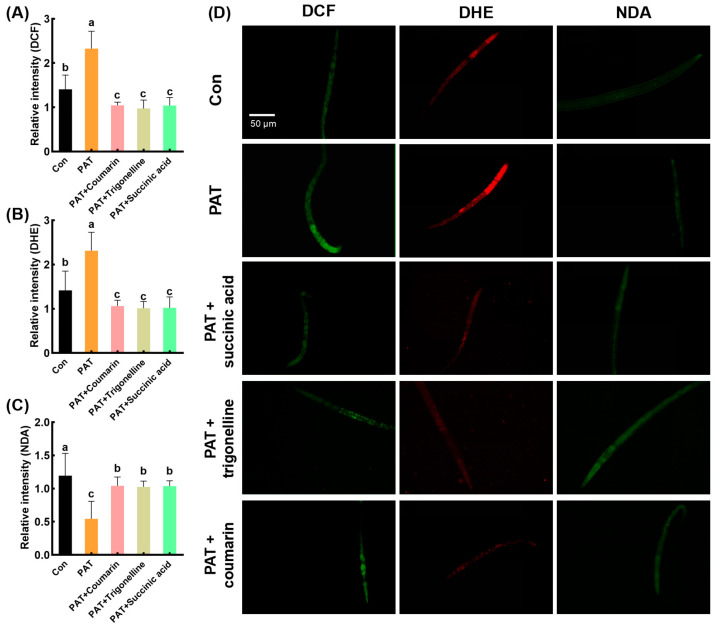
Alleviating effects of 3 active substances on oxidative damage induced by PAT in *C. elegans*. The relative fluorescence intensity quantification of *C. elegans* stained by DCF (**A**), DHE (**B**), and NDA (**C**) probes, and the representative images of the fluorescence staining observation (**D**). “Con” referred to the control group, and “PAT” referred to the model group treated with 50 µg/mL PAT. “PAT + Coumarin”, “PAT + Trigonelline”, and “PAT + Succinic acid” referred to groups treated with 50 µg/mL PAT and corresponding compound with concentrations of 100 µg/mL, respectively. Means with different letters within the same row differ significantly (*p* < 0.05).

Since coumarin, trigonelline, and succinic acid showed promising binding affinities to antioxidant-related proteins in docking simulation, the alleviating effects of which were further evaluated by determining rescue of locomotion parameters (Figure 5) and oxidative damage levels (Figure 6). In the 25 µg/mL coumarin treatment group, the frequency of body bends and head swings in nematodes increased compared to the PAT-treated group. The trigonelline group showed a dose-dependent effect in alleviating PAT toxicity, with higher concentrations resulting in more pronounced protective effects. At 100 µg/mL, the number of body bends and head swings were comparable to those in the blank control group. In contrast, the succinic acid treatment group showed limited effectiveness in alleviating PAT toxicity, as the frequency of body bends and head swings was not significantly different from that of the PAT-treated group. These results indicate that coumarin and trigonelline provided some protective effects against PAT-induced motility impairments, whereas succinic acid showed limited efficacy.

After treatment with succinic acid, trigonelline, and coumarin, the fluorescence intensities of the DCF and DHE probes were significantly reduced compared to the PAT-treated group (Figure 6A,B,D). Moreover, the fluorescence intensity of the NDA probe was significantly restored in all three treatment groups, showing a 1.90-, 1.88-, and 1.92-fold increase compared to the PAT-only treatment group, respectively (Figure 6D). These results suggest that these 3 compounds effectively protected the nematodes from PAT-induced oxidative stress.

These findings demonstrated that three compounds showing strong binding affinities in molecular docking simulations, especially coumarin and trigonelline, effectively alleviated PAT-induced toxicity in *C. elegans*. This mechanistic alignment validates that ligand-receptor binding energetics dictate antioxidant functionality in *C. elegans*.

## 4. Discussion

As a potent foodborne mycotoxin, PAT contamination poses significant health risks to both humans and livestock through dietary exposure. The primary toxicological mechanism involves PAT’s electrophilic properties, enabling covalent conjugation with cellular thiol groups in glutathione (GSH), cysteine, and thioglycolate [8]. This thiol depletion disrupts redox homeostasis, triggering excessive ROS generation [32]. Subsequent oxidative stress arises from the disruption of pro-oxidant/antioxidant equilibrium, exacerbating lipid peroxidation of polyunsaturated fatty acids and oxidative DNA modifications [9]. These molecular perturbations manifest as cellular dysfunction, ultimately contributing to systemic toxicity.

Current research emphasizes the prophylactic potential of phytochemical interventions against mycotoxin-induced oxidative damage. Natural compounds demonstrate dual mechanisms of action: ROS scavenging and upregulation of endogenous antioxidant defenses. For example, curcumin demonstrates protective effects against aflatoxin toxicity through modulation of the TLR1/RIPK signaling pathway, attenuation of oxidative stress biomarkers, and suppression of inflammatory cytokine production in avian hepatic tissues [14]. Similarly, anthocyanin supplementation has been shown to ameliorate zearalenone-induced hepatic oxidative stress in murine models by enhancing SOD and CAT activities [33]. Consistent with these findings, this study revealed that EEAT effectively mitigated PAT-induced oxidative damage (Figure 2) and enhanced SOD and GSH-ST enzyme activities (Figure 3), which contributed to the alleviation of *C. elegans* locomotory impairments (Figure 1).

*A. terricola* is a fungal species originally isolated from *Cordyceps gunnii*, and its fermentation products exhibit compositional parallels with those of the latter. As a microbial derivative widely utilized in animal feed, ATC has been shown to exhibit immunomodulatory and antioxidant properties across diverse animal models [18]. For instance, in a lipopolysaccharide (LPS)-induced mastitis rat model, ATC mitigated oxidative stress and inflammatory injury through suppression of the MAPK signaling pathway. This intervention significantly reduced levels of pro-inflammatory cytokines (TNF-α and IL-6) while concurrently elevating the activity of antioxidant enzymes, including SOD and GSH-Px [20]. Comparable effects were observed in weaned piglets and dairy cows, where ATC administration enhanced systemic redox balance by boosting antioxidant enzyme activities (T-AOC and CAT) and reducing malondialdehyde (MDA) accumulation [18,19]. Nevertheless, the molecular mechanisms underpinning ATC’s antioxidant effects remain incompletely elucidated, primarily due to insufficient characterization of its bioactive components and their interactions with cellular targets.

Traditional Chinese medicine (TCM) often relies on synergistic actions among multiple constituents and biological pathways. Recent advances in network pharmacology, particularly virtual docking-based approaches, have established the “component-target-disease” paradigm as a robust strategy for deciphering the mechanistic basis of herbal therapeutics [34,35]. Applying this framework, combined with metabolite profiling and molecular docking, could systematically unravel how EEAT counteracts PAT toxicity. As an efficient approach, UPLC-ESI-MS/MS analyses [36] were employed and have identified a spectrum of bioactive compounds in EEAT, such as succinic acid, coumarin derivatives, trigonelline, citric acid, jasmonic acid, and shikimic acid (Table 1 and Table 2). Contemporary pharmacological studies highlight the therapeutic potential of coumarin derivatives in oxidative stress-related pathologies through rational structural optimization [37,38]. Shikimic acid derivatives exhibit dual anti-inflammatory and antioxidant activities via modulation of AKT/Nrf2 and NF-κB signaling pathways [39,40]. Furthermore, citric acid demonstrates cytoprotective effects through antioxidant enzyme induction and immunomodulatory activity at the cellular level [41]. These identified compounds collectively contribute to the observed antioxidant capacity of EEAT.

Molecular docking analysis revealed strong binding affinities between EEAT components (coumarin, trigonelline, and succinic acid) and key nematode longevity regulators SIR-2.1 (−7.0 to −5.2 kcal/mol) and DAF-2 (−6.6 to −5.5 kcal/mol) (Figure 4, Table 3). SIR-2.1 modulates redox homeostasis through phase-dependent regulation of antioxidant gene expression [42,43,44], while DAF-2, the insulin/IGF-1 receptor homolog, critically regulates oxidative stress responses [45]. This aligns with previous findings that *L. plantarum* and *S. cerevisiae* mitigate PAT toxicity in *C. elegans* via IGF-1 pathway activation and antioxidant gene upregulation (e.g., sod-3, spp-2, and lys-2) [10,11]. Based on these findings, our results suggest that the major bioactive compounds in EEAT mitigate PAT toxicity by targeting key proteins involved in antioxidant-related pathways in *C. elegans*. To confirm this mechanism, future studies could test whether mutating specific binding sites in these proteins (e.g., key amino acids predicted by docking analysis) reduces toxicity alleviation effects. Such experiments would directly link molecular interactions to functional outcomes.

## 5. Conclusions

This study elucidates that PAT exposure induces oxidative stress-mediated neuromuscular dysfunction in *C. elegans*, characterized by 16% and 30% reductions in body bending and head swing frequencies, respectively. EEAT administration potently counteracted these toxic manifestations, normalizing motility parameters to control-equivalent levels while abolishing PAT-triggered ROS and O_2_^−^ (2.2- and 2.1-fold elevations). Concurrently, EEAT restored PAT-compromised antioxidant defenses, rescuing SOD and GSH-ST activities by 63.1% and 90.1% to near-homeostatic ranges. Molecular docking and experimental validation identified coumarin and trigonelline as pivotal bioactive constituents in EEAT, exhibiting strong binding affinities with antioxidant regulators SIR-2.1 and DAF-2. These findings contribute to the expanding knowledge of *Cordyceps* fungi and their potential applications in toxin mitigation and antioxidant research.

## Figures and Tables

**Table 1:** Compounds with level 1 confidence screened in negative ion mode.

RT (min) *	Name	*m/z*	Formula	Mass Errors (ppm)	RA
0.811	Lysine	104.03537	C_3_H_7_NO_3_	0.50	187,777,184.87
0.815	4-Hydroxyproline	130.05098	C_5_H_9_NO_3_	0.10	467,035,200.25
0.843	D-(-)quinic acid	191.05608	C_7_H_12_O_6_	−0.65	19,110,219.03
0.912	Uracil	111.02004	C_4_H_4_N_2_O_2_	0.39	62,055,885.23
0.953	Succinic acid	117.0194	C_4_H_6_O_4_	0.59	2,421,659,365.08
0.962	Shikimic acid	173.04547	C_7_H_10_O_5_	−0.72	10,718,161.54
1.014	Adenine	134.0473	C_5_H_5_N_5_	0.62	67,826,555.72
1.042	Citric acid	191.01973	C_6_H_8_O_7_	0.02	174,899,125.03
1.248	Leucine	130.08737	C_6_H_13_NO_2_	0.17	2,732,637,408.76

*: “RT” represented the retention time (min); “*m*/*z*” represented the mass-to-charge ratio; “mass errors” represented the difference between the actual mass of the metabolite and the mass of that in the database (ppm); “RA” represented the relative abundance.

**Table 2:** Compounds with level 1 confidence screened in positive ion mode.

RT (min) *	Name	*m*/*z*	Formula	Mass Errors (ppm)	RA
0.78	D-fructose-1,6-Diphosphate sodium	341.003	C_6_H_14_O_12_P_2_	0.27	8,309,708.86
0.944	Acetylcholine	146.118	C_7_H_15_NO_2_	1.36	287,983,209.06
0.946	Trigonelline	138.055	C_7_H_7_NO_2_	0.83	232,929,326.28
1.005	Guanine	152.05692	C_5_H_5_N_5_O	1.54	448,995,412.07
1.022	L-Isoleucine	132.10207	C_6_H_13_NO_2_	1.25	1,906,817,715.69
1.058	4-Hydroxyproline	132.06573	C_5_H_9_NO_3_	1.64	1,034,646,396.52
1.061	Adenine	136.06201	C_5_H_5_N_5_	1.78	1,162,426,530.13
1.131	Galactitol	183.08659	C_6_H_14_O_6_	1.53	759,773,486.42
1.158	2,2-Dimethylsuccinic acid	147.06535	C_6_H_10_O_4_	1.10	521,316,184.20
1.188	4-Guandinobutyric acid	146.09254	C_5_H_11_N_3_O_2_	0.92	109,412,261.72
10.414	Coumarin	147.04411	C_9_H_6_O_2_	0.39	114,026,419.58
12.834	Jasmonic acid	211.1329	C_12_H_18_O_3_	0.16	19,143,380.67
14.444	Cortisol	345.2059	C_21_H_30_O_5_	−0.20	29,494,327.46
19.314	Bilirubin	585.2713	C_33_H_36_N_4_O_6_	0.92	19,929,457.87
2.658	Isohexanoic acid	132.1021	C_6_H_13_NO_2_	1.46	1,618,895,399.44
3.869	L-Phenylalanine	166.08652	C_9_H_11_NO_2_	1.59	246,308,482.14

*: “RT” represented the retention time (min); “*m*/*z*” represented the mass-to-charge ratio; “mass errors” represented the difference between the actual mass of the metabolite and the mass of that in the database (ppm); “RA” represented the relative abundance.

**Table 3:** Optimization results of molecular docking.

Affinity (kcal/mol)			Ligand		
Accession Number		Protein	Coumarin	Trigonelline	Succinic Acid
Receptor	PDB ID: 4JDE	SKN-1	−5.6	−4.5	−3.9
	PDB ID: 3DC5	SOD-3	−5.2	−4.5	−3.9
	PDB ID: 3K62	GST-4	−5.9	−5.0	−4.4
	PDB ID: 2P32	HSP-1	−5.8	−5.4	−4.4
	UniProt: O16850	DAF-16	−5.1	−4.2	−3.6
	UniProt: G5EFT5	HSF-1	−5.7	−4.3	−4.1
	UniProt: Q968Y9	DAF-2	−6.6	−5.5	−4.2
	UniProt: Q94125	AGE-1	−6.5	−5.2	−4.5
	UniProt: P13508	GLP-1	−6.2	−4.9	−4.5
	UniProt: Q95ZQ4	AAK-2	−6.0	−4.7	−4.1
	UniProt: Q23272	ATFS-1	−5.0	−4.1	−3.3
	UniProt: P91302	UBL-5	−4.4	−3.9	−3.8
	UniProt: Q21921	SIR-2.1	−7.0	−4.4	−5.2
	UniProt: Q9U298	EAT-2	−5.7	−4.6	−3.9

## Data Availability

Data are contained within the article or Supplementary Material.

## Supplementary Materials

The following supporting information can be downloaded at: https://www.mdpi.com/article/10.3390/antiox14050509/s1, Figure S1: The chromatographic profile of the base peak ion in EEAT component analysis; Table S1: Grid Box parameters.
