# Supplementary material for: Alleviating Effects of Ethanol Extract from Acremonium terricola Culture on Patulin Toxicity
Source: Antioxidants (Basel). 2025 Apr 24;14(5):509. doi: 10.3390/antiox14050509 (PMC12108246; doi:10.3390/antiox14050509)
Supplement: Supplementary file 1 [file antioxidants-14-00509-s001.zip › antioxidants-3511515-supplementary.pdf]

Alleviating effects of ethanol extract from *Acremonium terricola* culture on patulin toxicity

Haiyan Lin[1, 2], Savindi Kaushalya Edirisinghe[1], Ijeoma Esther Okolo [1], Zhen Chen[1], Juan Sun[1], Wei Hong[3], Ruiyu Zhu[1]*

1: School of Biological and Chemical Engineering, Zhejiang University of Science and Technology, Hangzhou, 310023, China;

2: College of Food Science, Fujian Agriculture and Forestry University, Fuzhou, 350002, China;

3: Key Laboratory of Endemic and Ethnic Diseases, Ministry of Education and Key Laboratory of Medical Molecular Biology of Guizhou Province, Guizhou Medical University, Guiyang 550004, China;

*Corresponding: zhury15@zust.edu.cn (Ruiyu Zhu).

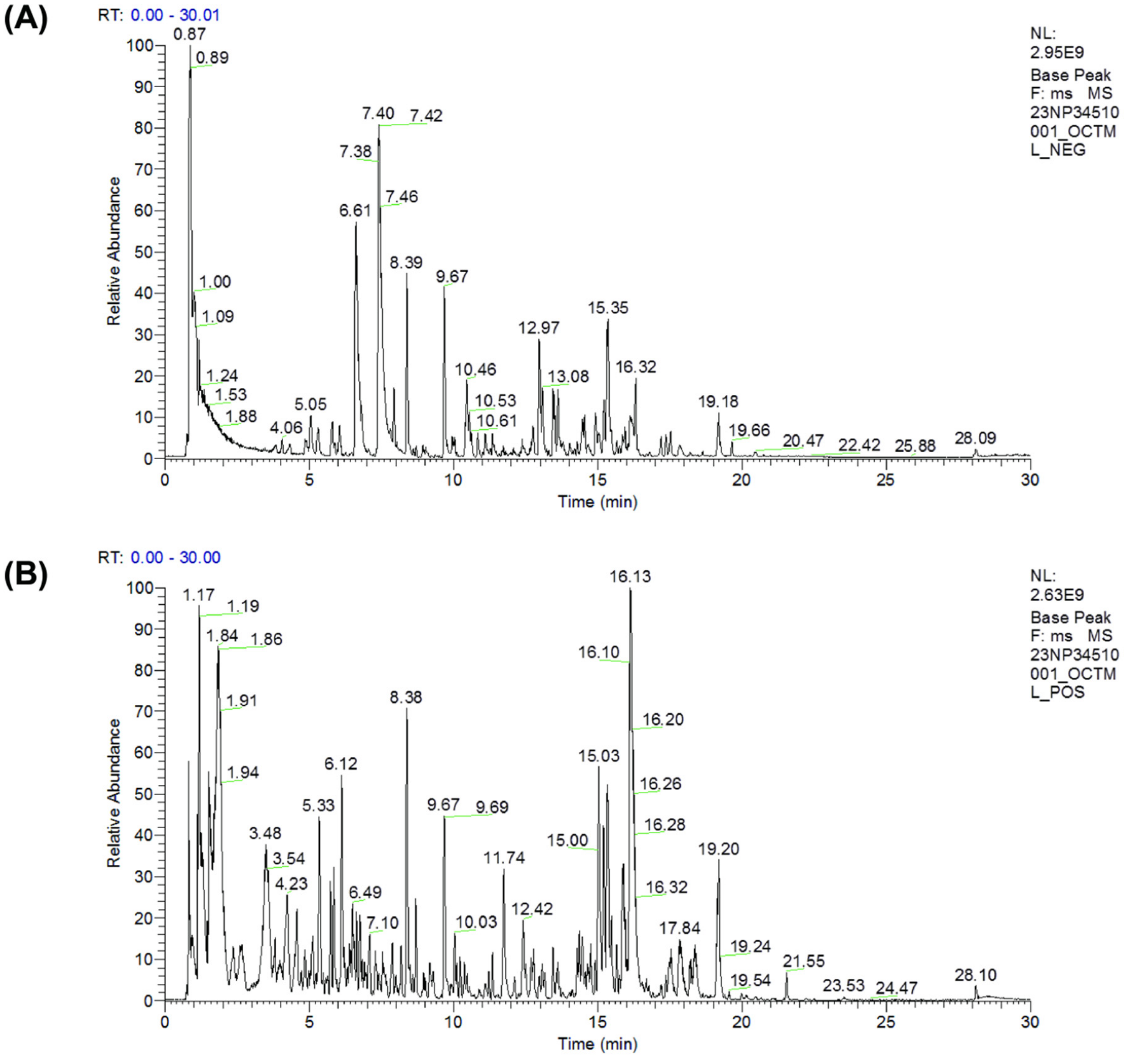

Figure S1. The chromatographic profile of the base peak ion in EEAT component analysis. (A) negative ions mode; (B) positive ions mode.

***Table S1.* Grid Box parameters**

Receptor	Center X	Center Y	Center Z	Size X	Size Y	Size Z
SKN-1	-26.787	-11.733	19.916	44.25	47.25	47.25
SOD-3	7.63	-0.417	0.752	67.989	60.956	66.817
GST-4	13.681	33.014	4.342	72.683	93.45	78.617
HSP-1	-0.032	-34.985	17.054	79.45	79.45	79.45
DAF-16	0	0	0	126	126	126
HSF-1	-18.696	-2.552	10.059	126	126	126
DAF-2	-4.84	0.601	-7.002	126	126	126
AGE-1	-0.075	2.544	-17.618	110	82	126
GLP-1	-7.409	6.231	-2.241	126	126	126
AAK-2	-6.48	6.931	-6.661	120	102	124
ATFS-1	3.106	1.808	-13.037	126	126	126
UBL-5	0.085	2.328	0.598	38.25	33.75	36
SIR-2.1	-4.122	-2.974	-5.756	126	120	124
EAT-2	-13.478	1.04	1.607	126	82	126
